# First report of novel single-nucleotide polymorphisms and genetic characteristics in the open reading frame of the prion protein gene (*PRNP*) in bats

**DOI:** 10.3389/fvets.2026.1777891

**Published:** 2026-05-13

**Authors:** Doan-Phuong-Anh Tran, Da-In Choi, Eun-Jee Na, Jae-Ku Oem, Byung-Hoon Jeong

**Affiliations:** 1 Korea Zoonosis Research Institute, Jeonbuk National University, Iksan, Jeonbuk, Republic of Korea; 2 Department of Bioactive Material Sciences, Jeonbuk National University, Jeonju, Jeonbuk, Republic of Korea; 3 Laboratory of Veterinary Infectious Diseases, College of Veterinary Medicine, Jeonbuk National University, Iksan, Jeonbuk, Republic of Korea

**Keywords:** bats, polymorphisms, prion disease, prion protein gene, PRNP, SNPs

## Abstract

**Background:**

Prion diseases are fatal neurodegenerative disorders capable of causing transmissible spongiform encephalopathies (TSEs) and have been reported in humans and several animal species. Genetic polymorphisms in the prion protein gene (*PRNP*) are known to influence the conformational conversion of the normal cellular prion protein (PrP^C^) into its pathogenic isoform (PrP^Sc^), thereby affecting disease susceptibility and pathogenesis. However, single-nucleotide polymorphisms (SNPs) in the *PRNP* gene have not been reported in bats, which remain free from prion disease reports despite their wide ecological diversity.

**Methods:**

We analyzed *PRNP* sequences from 94 bat samples and conducted comparative analyses with *PRNP* sequences from other animal species. The coding region of the bat *PRNP* gene was amplified and sequenced to identify genetic variants. Genotype, allele, and haplotype frequencies were calculated, and the potential functional and structural impacts of identified SNPs were predicted using *in silico* tools, including PolyPhen-2, PANTHER, SIFT, and SODA. Secondary and tertiary structures of bat PrP were modeled and visualized using SWISS-MODEL and Swiss-PdbViewer.

**Results:**

Five polymorphisms were identified in the bat *PRNP* gene, among which only one non-synonymous SNP, c.86A>G (K29R), was detected. Linkage disequilibrium and haplotype analyses revealed moderate genetic diversity in the bat *PRNP* locus. *In silico* prediction indicated that the K29R substitution is likely to be functionally neutral, without notable alterations in protein solubility or secondary structure. The modeled PrP structure of bats showed high overall conservation compared with other mammalian species.

**Conclusion:**

This study provides the first report describing *PRNP* genetic polymorphisms in bats, including the novel K29R variant. These findings offer essential insights into molecular evolution and possible mechanisms underlying natural resistance to prion diseases in bats.

## 1 Introduction

Prion diseases are classified as transmissible spongiform encephalopathies (TSEs) and are considered fatal disorders. The pathogenesis of prion diseases involves neurodegenerative processes triggered by the accumulation of the abnormal prion protein isoform (PrP^Sc^). These diseases are caused by the misfolding of the normal cellular prion protein (PrP^C^) into its abnormal isoform, leading to structural and functional alterations in the protein (1). Compared with the normal form, PrP^Sc^ adopts a β-sheet-rich conformation and exhibits decreased solubility in detergents (1). Initially, scrapie was described as a spongiform encephalopathy in sheep (2), and subsequently, various prion diseases have been identified in humans such as sporadic Creutzfeldt–Jakob disease (CJD), familial CJD, fatal familial insomnia (FFI), Gerstmann-Sträussler-Scheinker disease (GSS), iatrogenic CJD and Kuru, as well as in a diverse range of host species, including transmissible mink encephalopathy (TME) in mink, bovine spongiform encephalopathy (BSE) in cattle, chronic wasting disease (CWD) in elk and deer, camel prion disease (CPD) in camels, and feline spongiform encephalopathy (FSE) in cats (3–8).

Previous research has revealed that polymorphisms in the prion protein gene (*PRNP*) profoundly influence the transformation of PrP^C^ into PrP^Sc^ and the pathogenesis of prion diseases. Such genetic variations also affect PrP expression levels, thereby modulating host susceptibility or resistance. In humans, the non-synonymous single-nucleotide polymorphism (SNP) at codon 129 encodes either methionine or valine, and the SNP at codon 219 encodes either glutamic acid or lysine; both are strongly associated with prion disease susceptibility (4). Notably, all patients with variant CJD consistently exhibit methionine homozygosity at codon 129 (9). Moreover, Asian sporadic CJD patients pre-dominantly exhibit homozygosity for glutamic acid at codon 219, while no heterozygous or homozygous lysine polymorphisms are detected, supporting a protective effect of this residue against disease onset (10, 11).

In sheep, multiple non-synonymous SNPs have been identified in *PRNP*, including M112T, A136V, M137T, S138N, L141F, R151C, R154H, Q171R/H, N176K, and R211Q (2, 12–14). Among them, polymorphisms at codons 154 and 171, located within the C-terminal region, are associated with resistance to scrapie. Three key SNPs (A136V, R154H, and Q171R/H) give rise to five major haplotypes (ARR, AHQ, ARQ, ARH, and VRQ) that differ in disease susceptibility; ARR and AHQ confer resistance, whereas ARQ, ARH, and especially VRQ increase susceptibility (15). Similarly, several SNPs identified in goats (I142M, H143R, N146S, R211Q, and Q222K) reduce the risk of developing scrapie (15–18). Certain mammalian species also exhibit natural resistance to prion diseases due to specific amino acid residues in their PrP sequence. For instance, D159 in dogs and S167 in horses are thought to stabilize the PrP structure and prevent conversion to the pathogenic form (19, 20).

In addition to SNPs, insertion/deletion (indel) polymorphisms in the regulatory region of the *PRNP* have also been implicated in susceptibility to prion diseases. In cattle, such indels are known to affect *PRNP* expression levels and incubation periods, thereby influencing the risk of developing BSE (21, 22). Moreover, the promoter region with 23 bp and 12 bp indel polymorphisms has been shown to be associated with greater vulnerability to BSE (23).

Species-specific differences in *PRNP* expression and sequence composition are key determinants of prion susceptibility or resistance. Horses, for example, are known to be prion-resistant, displaying a phenomenon termed “non-adaptive prion amplification”. In both *in vitro* protein misfolding cyclic amplification and *in vivo* mouse models expressing equine PrP^C^, horse-derived prions failed to transmit disease, despite retaining infectivity toward their native host (24). Likewise, transgenic mouse studies have indicated that substitution at codon 163 of the canine *PRNP* gene confers strong resistance to prion infection; unlike humans and other susceptible species, dogs naturally harbor aspartic or glutamic acid at this site, instead of asparagine, which is believed to underlie their prion resistance (25).

Bats are evolutionarily unique as the only mammals able to perform sustained flight, distinguishing them from avian species (26). They are also recognized as natural reservoirs for a large range of zoonotic viruses, including coronaviruses, filoviruses, and lyssaviruses, many of which can induce severe neurological diseases in humans (27, 28). Among these, Nipah virus, first identified in 1998, is particularly notable for its ability to cause severe and often fatal encephalitis (29), and more recently, a bat-origin coronavirus triggered a global pandemic with devastating consequences (30). Given their ecological diversity and potential role in zoonotic transmission, investigating prion-related genetic variation in bats may provide new insights into interspecies barriers and molecular mechanisms of resistance to prion diseases.

In this study, we amplified the open reading frame (ORF) of the *PRNP* in 94 bat samples to identify potential genetic polymorphisms. We calculated genotype, allele, and haplotype frequencies and modeled the secondary and tertiary structures of PrP based on the identified non-synonymous SNPs using SWISS-MODEL and Swiss-PdbViewer (31–33). Furthermore, we performed *in silico* functional analyses to predict the structural and pathogenic implications of these SNPs.

## 2 Materials and methods

### 2.1 Sample preparation

Bat tissue samples were donated by the College of Veterinary Medicine at Jeonbuk National University; these samples had been preserved in a deep freezer (−80 °C). All experimental guidelines were approved by the Institutional Animal Care and Use Committee (IACUC) of Jeonbuk National University (NON2023–227).

### 2.2 Genetic characteristics analysis of the *PRNP* in bats

To analyze the *PRNP* gene of bats, polymerase chain reaction (PCR) was performed to amplify the ORF region using the following primers: *PRNP*-F (CCCTCTTCATTTTGCAGATAAGCC) and *PRNP*-R (CGGCCTTCTCTTTCCACCAT). Detailed information on primer binding sites and the PCR amplicon size (773 bp) is provided in Supplementary Table S1. BioFACT™ Taq DNA Polymerase (Biofact, Daejeon, Korea) was used for PCR, and the PCR mixture (25 μl total volume) contained 2.5 μl of 10 × *Taq* reaction buffer, 1 μl of 10 mM dNTP mix, 1 μl of each primer (10 μm), 2.5 μl of 5 × band helper, 0.2 μl of *Taq* DNA polymerase, and distilled water. The PCR was carried out under the following thermal cycling conditions: an initial denaturation at 95 °C for 2 min; 35 cycles of denaturation at 94 °C for 45 s, annealing at 63 °C for 45 s, and extension at 72 °C for 1 min; followed by a final extension at 72 °C for 5 min. Agarose gel electrophoresis was performed to detect PCR products of the expected size (Supplementary Figure S1). The PCR products were purified using a FavorPrep GEL/PCR Purification Mini Kit (FAVORGEN, Pingtung City, Taiwan). The purified PCR products were subjected to Sanger sequencing using an ABI 3730xl DNA analyzer (Applied Biosystems, Foster City, CA, USA), and the sequencing reactions were performed by a commercial sequencing service provider. The resulting chromatograms were analyzed using FinchTV software (Geospiza Inc., Seattle, WA, USA) for SNP identification.

### 2.3 Phylogenetic analysis and alignments of the PrP amino acid sequence between bats and a range of other species

To assess the sequence similarity of PrP among various species, we collected the published amino acid sequences from GenBank at the National Center for Biotechnology Information (NCBI) including human (NP_000302.1) as well as other species such as cattle (NP_851358.2), cat (XP_019682354.2), dog (NP_001013441.1), raccoon dog (XP_055159453.1), rabbit (NP_001075490.1), horse (NP_001137270.2), sheep (NP_001009481.1), goat (NP_001301176.1), and chicken (NP_990796.2). A phylogenetic tree was constructed to analyze the evolutionary relationships of the *PRNP* gene between bats and the aforementioned species with Mega12 software (34). Clustal Omega was employed to perform multiple sequence alignments, enabling the comparison of amino acid sequence lengths and the assessment of sequence conservation among the *PRNP* sequences of humans, previously reported species, and bats (35).

### 2.4 Prediction of the PrP structure

The three-dimensional (3D) structures of the PrPs were constructed by ColabFold v1.5.5: AlphaFold2 (36, 37). ColabFold included three steps, in which the first step involved building multiple sequence alignments (MSAs) based on MMseqs2 homology searches. Then, the output was assessed by MSA depth and diversity and by AlphaFold2 confidence measures. The tertiary protein structures were visualized with Swiss PDB Viewer 4.1, and the secondary structure pictures were drawn in PowerPoint using PDB data from ColabFold v1.5.5: AlphaFold2.

### 2.5 Evaluation of the effects of non-synonymous SNPs in the bat PrP *in silico*

Five different programs were used to predict whether the non-synonymous SNPs impact the structural or functional properties of bat PrP. PolyPhen-2 (http://genetics.bwh.harvard.edu/pph2/) predicts whether an amino acid substitution affects protein stability and functional properties. The software evaluates the potential impact of mutations based on structural features and comparative evolutionary considerations (38). PANTHER (http://www.pantherdb.org/tools/csnpScore.do) is a tool to investigate the potential deleterious effect of non-synonymous SNPs on the host via the conservation of evolutionary information, and its results can be used to estimate functional impact (39). SIFT (https://siftdna.org/) predicts the mutation's effect on protein function (40). SODA (http://old.protein.bio.unipd.it/soda/) was used to test the change in protein solubility based on the amino acid aggregation propensity and hydrophobicity (41). When Δ*S*_*Aggregation*_ > 0, the variants are predicted to increase solubility. Similarly, the protein's solubility also increases when Δ*S*_*Disoder*_ > 0 due to higher intrinsic disorder.

### 2.6 Statistical analysis

The Hardy-Weinberg equilibrium (HWE) test using Michael H. Court's calculator was conducted to evaluate whether the genotype frequencies in the selected sample conform to HWE and assess the stability of allele or genotype frequencies in the given population. A *P*-value of less than 0.05 suggests that the obtained genotyping data deviates from the HWE. This deviation may be attributed to factors such as mutations, selection, population structure, or genotyping errors. The linkage disequilibrium (LD) and haplotype distributions were assessed with Haploview version 4.2 (Broad Institute, Cambridge, MA, USA) (42). The LD analysis assesses the statistical associations among SNPs within each gene locus and evaluates the correlation between pairs of genetic loci, using the *r*^2^ and D values.

## 3 Results

### 3.1 Identification of the *PRNP* SNPs in bats

**Figure 1:**
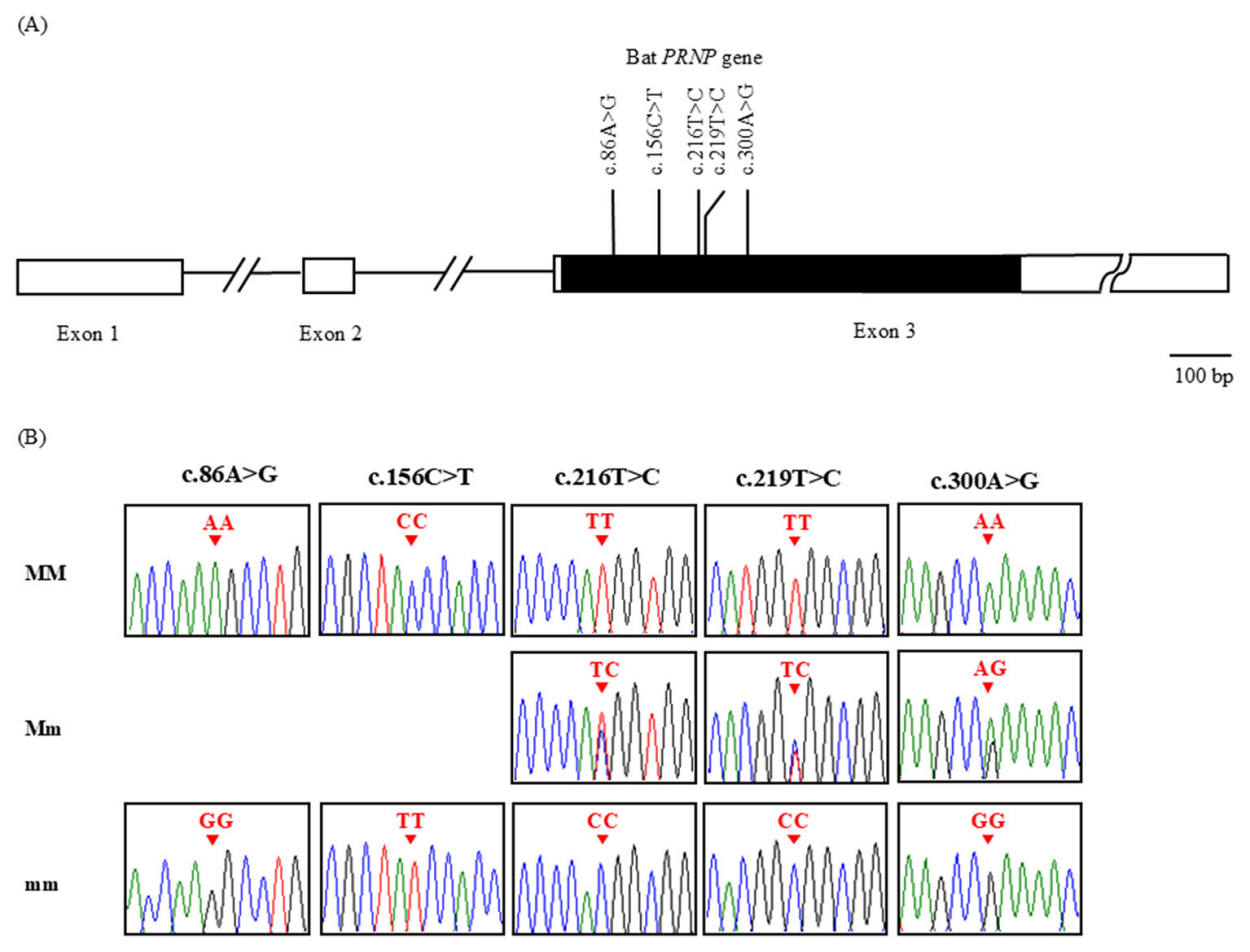
Identification of novel single-nucleotide polymorphisms (SNPs) in the bat prion protein gene (*PRNP*). **(A)** Schematic map of the bat *PRNP* showing the locations of the identified SNPs. **(B)** Electropherograms of the five novel SNPs detected in the *PRNP* gene from 94 bats. MM indicates the major homozygous genotype, Mm indicates the heterozygous genotype, and mm indicates the minor homozygous genotype. The colors of the peaks represent each DNA base (green: adenine; red: thymine; blue: cytosine; and black: guanine). Red arrows indicate the locations of the SNPs found in this study.

The *PRNP* gene in bats consists of three exons, with the ORF located in exon 3 and spanning 747 bp. We analyzed the ORF to identify genetic polymorphisms in the bat *PRNP* and found five novel SNPs: c.86A>G (K29R), c.156C>T (Y52Y), c.216T>C (H72H), c.219T>C (G73G), and c.300A>G (P100P). All identified SNPs were located within the ORF, and only c.86A>G (K29R) represented a non-synonymous substitution (Figure 1). The genotype and allele frequency results for these five SNPs are summarized in Table 1. In addition, LD analysis was performed to evaluate the extent of genetic linkage among five SNPs in the *PRNP* gene of bats; strong LD (*r*^2^ > 0.333) was observed between c.86A>G and c.156C>T, between c.216T>C and c.219T>C, between c.216T>C and c.300A>G, and between c.219T>C and c.300A>G (Table 2). Furthermore, haplotype analysis of the *PRNP* polymorphisms in bats revealed five major haplotypes, as shown in Table 3. The most frequent haplotype was ACTTA (55.8%), whereas GTTTA had the lowest frequency (1.1%) in the bat *PRNP*.

**Table 1:** Genotype and allele frequencies of the prion protein gene (*PRNP*) polymorphisms in 94 bats.

Polymorphisms	Genotype frequencies, *n* (%)			Allele frequencies, *n* (%)		HWE
	MM	Mm	mm	M	m	
c.86A>G	93 (98.9)	0 (0)	1 (1.1)	186 (98.9)	2 (1.1)	< 0.01
c.156C>T	93 (98.9)	0 (0)	1 (1.1)	186 (98.9)	2 (1.1)	< 0.01
c.216T>C	25 (26.6)	60 (63.8)	9 (9.6)	110 (58.5)	78 (41.5)	< 0.01
c.219T>C	26 (27.7)	61 (64.9)	7 (7.4)	113 (60.1)	75 (39.9)	< 0.01
c.300A>G	30 (31.9)	56 (59.6)	8 (8.5)	116 (61.7)	72 (38.3)	< 0.05

MM, Major homozygote; Mm, Heterozygote; mm, Minor homozygote; M, Major allele; m, Minor allele. HWE, Hardy-Weinberg equilibrium.

**Table 2:** Linkage disequilibrium (LD) analysis among *PRNP* polymorphisms in bats.

Polymorphisms	c.86A>G	c.156C>T	c.216T>C	c.219T>C	c.300A>G
c.86A>G	—				
c.156C>T	**1.0**	—			
c.216T>C	0.008	0.008	—		
c.219T>C	0.007	0.007	**0.829**	—	
c.300A>G	0.007	0.007	**0.789**	**0.655**	—

Bold text indicates strong LD (r^2^ >0.333).

**Table 3:** Haplotype frequencies among five *PRNP* polymorphisms in bats.

Haplotype	Frequency, *n* (%)
ACTTA	105 (0.558)
ACCCG	64 (0.340)
ACCCA	8 (0.043)
ACCTG	6 (0.032)
GTTTA	2 (0.011)
Others^*^	3 (0.016)
Total	188 (1)

^*^Others contain rare haplotypes with frequencies of < 0.01.

### 3.2 Phylogenetic analysis, octapeptide repeat regions, and amino acid sequence alignments of PrP across species with comparison to bats

**Figure 2:**
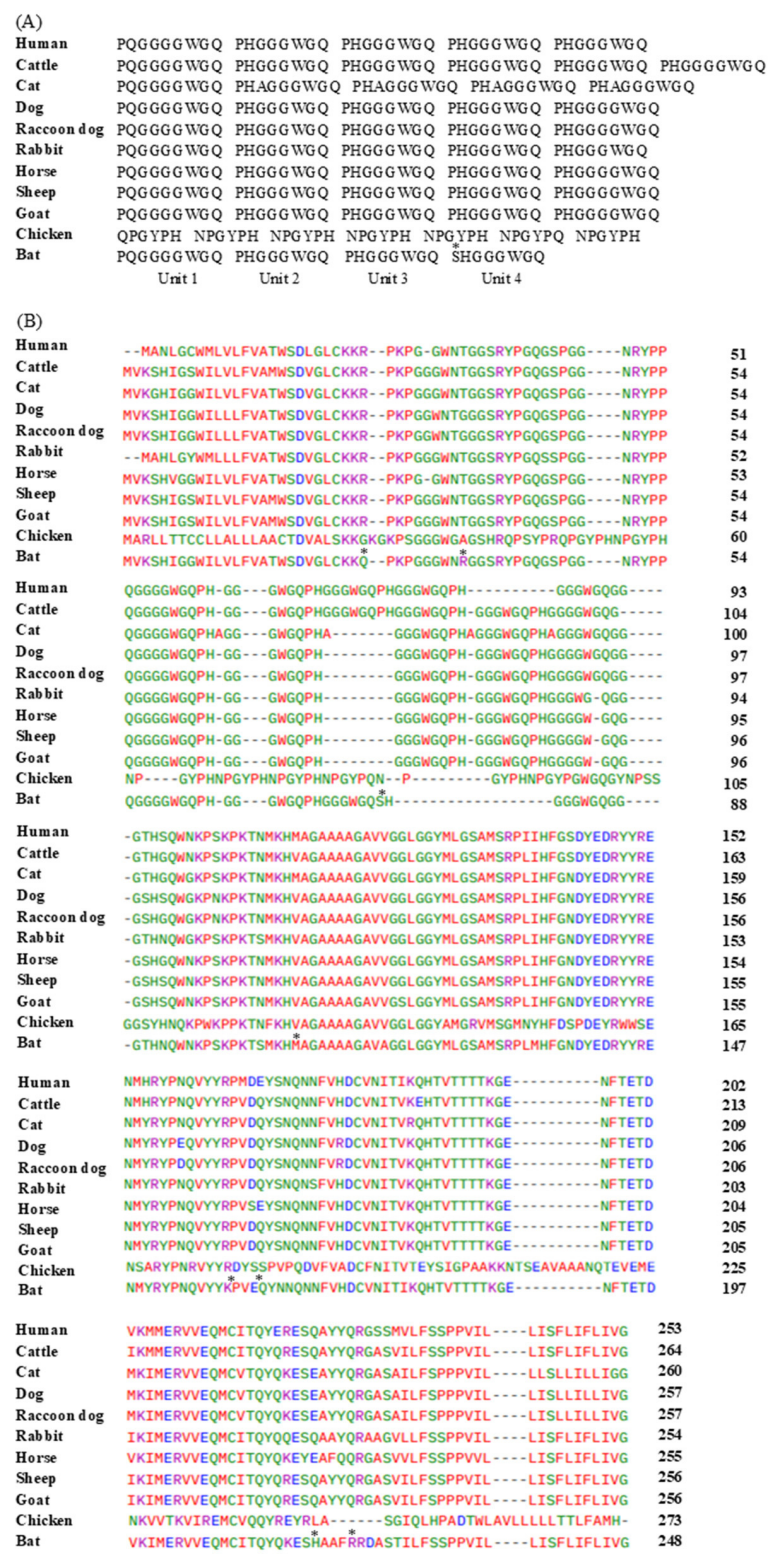
Amino acid sequences of prion protein (PrP) in bats and other representative species. **(A)** Comparisons of the octapeptide repeat regions among humans, bats, and other species. **(B)** Multiple sequence alignment of PrP amino acid sequences across species. Colors indicate the chemical properties of amino acids (blue: acidic; red: small and hydrophobic; magenta: basic; and green: hydroxyl, sulfhydryl, amine, and glycine). Asterisks indicate amino acid residues unique to bats.

The phylogenetic analysis of the PrP among several species shows that bats (*Rhinolophus ferrumequinum*) form a distinct lineage within the mammalian tree (Supplementary Figure S2). Bats cluster more closely with carnivorous mammals, forming a branch proximal to dogs (*Canis lupus familiaris*), raccoon dogs (*Nyctereutes procyonoides*). This clustering suggests evolutionary conservation of *PRNP* between bats and prion-resistant species. Besides, we compared the tandem repeat region (octapeptide repeats in most mammals and hexapeptide repeats in chickens) among humans, cattle, cats, dogs, raccoon dogs, rabbits, horses, sheep, goats, chickens, and bats (Figure 2A). While the bat *PRNP* (PHAGGGWGQ) contained one fewer repeat unit compared with the human *PRNP*, the chicken PrP, which consists of a six-amino acid repeat (QPGYPH), was strikingly different. The repeat units of bats were largely identical to those of other mammals. However, the fourth repeat unit in bats was slightly different from the consensus sequence (SHGGGWGQ instead of PHGGGWGQ). In addition, we used Clustal Omega to analyze the evolutionary relationships of PrP protein sequences among humans, cattle, sheep, goats, horses, dogs, cats, raccoon dogs, chickens, and bats (Figure 2B). The results of the multiple sequence alignment indicated that the bat PrP protein, comprising 248 amino acids, was shorter than those of other mammals (cat: 260 aa; dog: 257 aa; sheep: 256 aa; human: 253 aa) and the chicken PrP (273 aa). Moreover, a few specific amino acid residues were uniquely present in bats, including Q27, R36, S79, M132, K159, E162, H219, and R222.

### 3.3 Prediction of the secondary and tertiary protein structures among multiple different species

**Figure 3:**
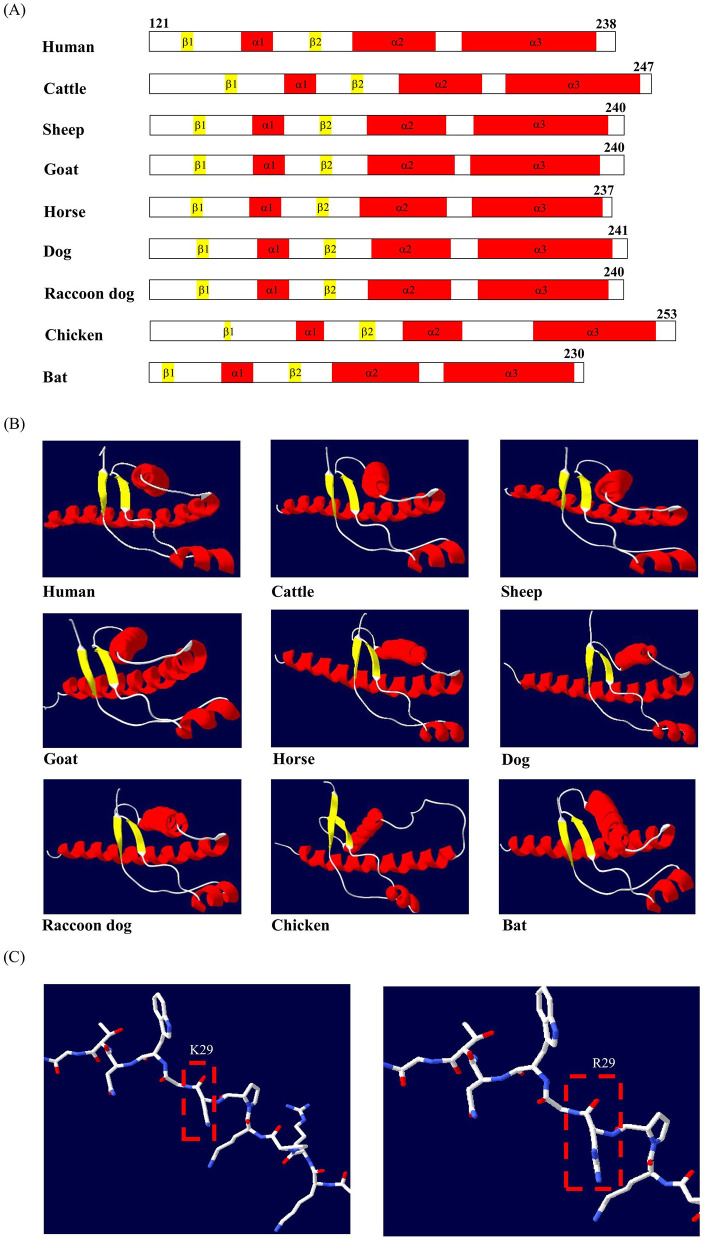
Secondary and tertiary structures of prion proteins (PrPs) from various species. **(A)** Predicted secondary structures of PrPs such as humans, cattle, sheep, goats, horses, dogs, raccoon dogs, chickens, and bats. **(B)** Predicted tertiary structures of the corresponding PrPs. Colors represent α-helices (red), β-sheets (yellow), and coils (white). **(C)** Comparison of the 3D structure according to the non-synonymous SNP K29R alleles.

To visualize the predicted PrP structures, we used the output from ColabFold to generate secondary structures and compare the structural similarities among various species (Figure 3A). All species, including humans, cattle, sheep, goats, horses, dogs, cats, rabbits, raccoon dogs, and chickens, exhibited highly similar PrP structures. The bat PrP protein in the structured region also showed a comparable folding pattern, containing three α-helical regions (residues 139–147, 167–189, and 195–228) and two β-sheet regions (residues 124–126 and 156–158). The 3D models were generated using Swiss PDB Viewer to visualize the 3D conformation of the amino acid residues (Figure 3B). The tertiary structures in the structured region of humans, other mammalian species, and bats all consisted of three α-helices and two β-sheets. Moreover, we investigated changes in hydrogen bonds (H-bonds) caused by the non-synonymous SNPs and their interactions with neighboring residues. No H-bond formation was observed between amino acid residue 29 and the surrounding residues in either the wild-type or the non-synonymous variant (Figure 3C).

### 3.4 Comparisons of the numbers and distributions of polymorphisms between different species based on *PRNP*

**Figure 4:**
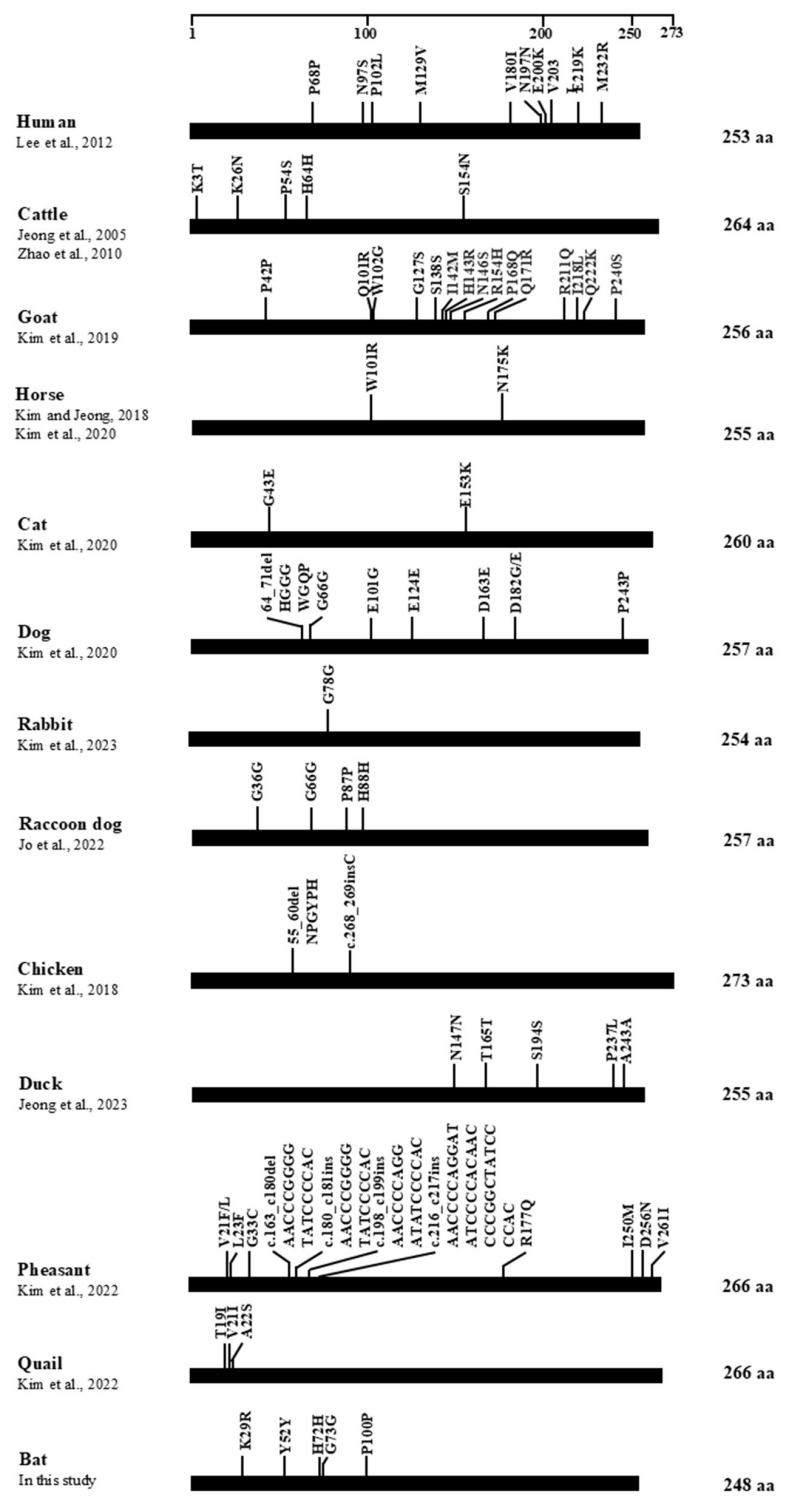
Map of single-nucleotide polymorphisms (SNPs) in the open reading frame (ORF) of the *PRNP* gene across various species. The edged horizontal bar represents the amino acid length of the *PRNP*. The figure illustrates previously reported genetic polymorphisms of *PRNP* in humans and multiple species including humans (48), cattle (49, 50), goats (51), horses (52, 53), cats (54), dogs (55), rabbits (56), raccoon dogs (57), chickens (58), ducks (59), pheasants (60), and quails (61), as well as bats (reported in this study).

To compare *PRNP* similarity across humans and various species in terms of SNP number and location, we compiled SNP data from previous reports (Figure 4). The SNP distribution in bats was distinctly different from that of other species. Notably, among the five SNPs identified in bats, no deletion or insertion polymorphisms were observed. Compared with other mammals, such as humans, goats, and pheasants, which harbor numerous non-synonymous mutations in the C-terminal domain associated with disease susceptibility or resistance, the bat *PRNP* exhibited a markedly lower level of genetic diversity. This relatively conserved pattern suggests that the *PRNP* gene in bats may be under strong purifying selection pressure, reflecting a functional constraint to maintain PrP stability. Moreover, the absence of indel polymorphisms within the octapeptide-like repeat region indicates that structural alterations in the N-terminal domain are rare in bats, contrasting with the frequent insertions or deletions observed in prion-susceptible mammals such as humans and cattle.

### 3.5 *In silico* analysis of the influence of non-synonymous SNPs on PrP structural and functional properties in bats

Genetic variations can result in amino acid substitutions that alter the functional, structural, and biochemical properties of proteins. To evaluate the potential deleterious effects of non-synonymous SNPs on structural integrity and functional characteristics of the bat *PRNP* gene, we employed five *in silico* programs, including PolyPhen-2, PANTHER, SNPs&GO, SIFT, and SODA, and analyzed their output data (Table 4). Among the five novel polymorphisms identified in the bat *PRNP*, only one non-synonymous SNP, c.86A>G (K29R), was detected. The available web-based tool, PolyPhen-2 indicated that this non-synonymous SNP could be “possibly damaging” to the functional aspect of the bat PrP protein, while the prediction by PANTHER demonstrated that this variant was probably benign with a score of 0.184. In addition, PANTHER predicts the potential pathogenicity of non-synonymous SNP variants by utilizing evolutionary conservation, which traces how long a particular amino acid has been conserved in a phylogenetic tree. This method helps assess the impact of variants on pathogenesis. According to the SNPs&GO tool, the only bat non-synonymous SNP was shown to be neutral with a score of 0.276. Another tool, SIFT, predicts the protein function affected by the change of an amino acid in the chain. A score < 0.05 indicates that this variant has a deleterious effect. In bat PrP, K29R had a score of 0.07, indicating “not deleterious”. In addition, the protein solubility affected by the amino acid sequence was predicted by SODA, which gave a score of 0, indicating “no significant change”.

**Table 4:** *In silico* investigation of non-synonymous single-nucleotide polymorphism effects in bats.

Polymorphism	Method	Score	Prediction
c.86A > G	PolyPhen-2	0.659	Possibly damaging
(K29R)	PANTHER	0.184	Probably benign
	SNPs & GO	0.276	Neutral
	SIFT	0.07	Not deleterious
	SODA	0	Neutral

## 4 Discussion

No cases of transmissible prion infection have been reported in bats. Various factors may contribute to the prion resistance observed in different species, among which genetic polymorphisms have been reported to modulate susceptibility to prion diseases (15–23). Hence, we conducted PCR amplification of the bat *PRNP* ORF and investigated its genetic polymorphisms. Notably, we identified five novel SNPs within this region, including one non-synonymous SNP (Figure 1 and Table 1). Interestingly, all SNPs in bats significantly deviated from HWE (*P* < 0.05) (Table 1). Furthermore, LD analysis based on *r*^2^ values across all polymorphisms revealed four pairs in strong LD (*r*^2^ >0.333), namely c.86A>G and c.156C>T; c.216T>C and c.219T>C; c.216T>C and c.300A>G; and c.219T>C and c.300A>G (Table 2). Both r^2^ and D' measures are sensitive to allele frequencies and missing haplotypes. Notably, low-frequency alleles can produce low r^2^ values despite actual linkage, emphasizing the importance of accounting for allele frequency in such studies (42). The *PRNP* gene consists of three exons, with the ORF located entirely within exon 3, which encodes the functional prion protein. Therefore, the present study focused on exon 3 to identify polymorphisms that may directly affect the amino acid sequence and structural properties of the prion protein. We acknowledge that intronic and other non-coding regions may contain important regulatory variants that could influence gene expression and disease susceptibility. However, this study was designed to prioritize variants with potential functional relevance at the protein level. Future studies will include comprehensive analyses of promoter and intronic regions to provide a more complete understanding of *PRNP* genetic variation.

Indel polymorphisms within the octapeptide repeat section of the *PRNP* gene have previously been reported to produce a pathogenic human PrP mutant (43, 44). The octapeptide repeat region of PrP is recognized as the primary site for copper binding, which is believed to be essential for the protein's physiological function (45, 46). In addition, the bat *PRNP* gene possesses a distinct repeat region consisting of four repeat units, which is unique compared to those of other species. Therefore, this feature may affect copper-binding capacity as well as the structural stability of PrP. Additional research is required to clarify the biological consequences of a decreased number of octapeptide repeats in bat PrP.

The molecular structure of PrP^C^ reveals an N-terminal signal sequence, an unstructured flexible region, and a well-defined structured C-terminal domain (47). Next, to examine structural conservation, the secondary and tertiary structures of bat PrP were analyzed. The bat protein in the globular domain was predicted to consist of three α-helices and two β-sheet domains (Figure 3). The overall structural composition of bat PrP was highly similar to that of other mammalian species, with the exception of chickens, known to be prion-resistant animals, which have a PrP structure that includes four α-helices and two β-sheet domains. In addition, the single non-synonymous SNP in bats did not induce any rearrangement in protein folding or formation of additional H-bonds involving nearby amino acid residues (Figure 3C). Therefore, both the conserved structural architecture of bat PrP and the lack of significant conformational changes caused by the non-synonymous SNP suggest that these features may not substantially affect prion susceptibility.

Based on SNP comparisons across species (Figure 4), the distribution and number of SNPs in bats did not follow the conserved patterns observed in mammals or birds, suggesting that these variations may have arisen as random mutations. Notably, the SNPs identified in bats exhibited a distinct distribution, appearing independently rather than following the evolutionary conservation trends typically seen in other species. Furthermore, multiple sequence alignment revealed that bats possess 8 unique amino acid residues, which may contribute to their distinctive PrP biochemical properties (Figure 2B). Taken together, the presence of randomly distributed SNPs and bat-specific amino acid residues may represent potential molecular factors underlying the apparent absence of prion infection in bats. Nonetheless, further experimental validation is required to determine the functional relevance of these SNPs and their possible protective effects against prion disease.

Finally, we conducted *in silico* analyses using prediction tools such as PolyPhen-2, PANTHER, SNPs&GO, SIFT, and SODA to evaluate the potential impact of the non-synonymous SNP (K29R) on PrP in bats (Table 4). PolyPhen-2 predicted that the bat PrP variant would be “probably damaging”. However, the functional consequences of this non-synonymous SNP and its potential association with prion disease susceptibility in bats require further experimental validation.

## 5 Conclusion

In conclusion, we identified five novel SNPs in the *PRNP* gene of bats, including a single non-synonymous SNP. We also calculated the genotype and allele frequencies of these variants. In addition, strong LD and haplotype analyses revealed five major haplotypes. Comparative analysis of the tandem repeat region of bat PrP with those of other species highlighted distinct differences in both sequence composition and the number of repeat units. The non-synonymous SNP (K29R) showed no detectable effect on the functional or structural properties of PrP, suggesting that this substitution may not alter prion protein behavior in bats.

## Data Availability

The datasets generated and/or analyzed during the current study are available in GenBank (Accession number: PX655145.1).
