# Supplementary material for: First report of novel single-nucleotide polymorphisms and genetic characteristics in the open reading frame of the prion protein gene (PRNP) in bats
Source: Front Vet Sci. 2026 May 13;13:1777891. doi: 10.3389/fvets.2026.1777891 (PMC13214133; doi:10.3389/fvets.2026.1777891)
Supplement: Supplementary file 1 [file Data_Sheet_1.docx]

**Supplementary Table S1.** PCR conditions and primer information for amplification of the open reading frame (ORF) of the *PRNP* gene in bats.

| **PCR Characteristics** | **Detail** |
| --- | --- |
| **PCR amplicon size** | 773 bp |
| **Sense primer sequence** | CCCTCTTCATTTTGCAGATAAGCC |
| **Antisense primer sequence** | ATGGTGGAAAGAGAAGGCCG |
| **PCR sequence** | CCCTCTTCATTTTGCAGATAAGCCATC**ATGGTGAAAAGCCACATAGGCGGCTGGATCCTGGTTCTCTTTGTGGCCACTTGGAGTGACGTGGGCCTCTGCAAGAAGCAACCAAAGCCTGGAGGAGGATGGAACCGTGGCGGGAGCCGATACCCGGGACAGGGCAGTCCTGGAGGCAACCGCTACCCACCCCAGGGCGGTGGCGGCTGGGGTCAGCCCCACGGCGGCGGCTGGGGTCAGCCCCATGGTGGCGGCTGGGGACAGTCCCATGGCGGAGGCTGGGGTCAAGGTGGTGGCACCCACAATCAGTGGAACAAGCCCAGTAAGCCAAAAACCAGCATGAAGCACATGGCAGGAGCTGCTGCGGCGGGGGCCGTGGCCGGGGGCCTCGGCGGCTACATGCTGGGGAGTGCCATGAGCAGGCCCCTCATGCATTTCGGCAACGACTATGAGGACCGTTATTATCGTGAAAACATGTACCGTTACCCCAACCAAGTGTACTACAAGCCCGTGGAGCAGTACAACAACCAGAACAACTTTGTGCATGACTGCGTCAACATCACCATCAAGCAGCACACTGTCACCACCACCACCAAGGGGGAGAACTTCACTGAGACCGACGTCAAGATAATGGAGCGCGTGGTGGAACAGATGTGTATCACCCAGTACCAGAAAGAGTCCCATGCAGCTTTCCGAAGGGATGCGAGTACGATCCTCTTCTCCTCCCCTCCAGTGATCCTGCTCATCTCTTTCCTCATTTTCCTAATAGTGGGATGA**GGCCGGCCTTCTCTTTCCACCAT |

Underlined texts indicate the primer binding sites. Bold text indicates the open reading frame of the *PRNP* gene in bats.


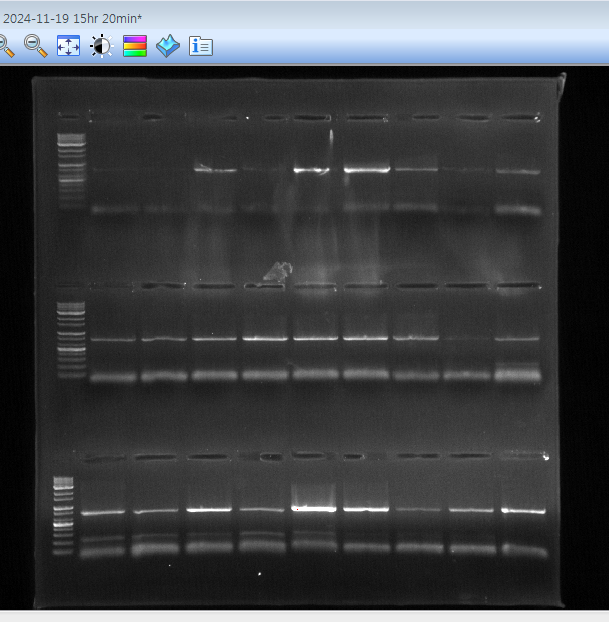


M 1 2 3 4 5

773 bp

1000 bp

500 bp

**Supplementary Figure S1.** Agarose gel electrophoresis of PCR products amplified from the *PRNP* open reading frame (ORF) in bats. The expected size of PCR products is 773 bp. A DNA ladder (M) was used as a molecular size marker. Lanes 1-5 represent individual samples.

**
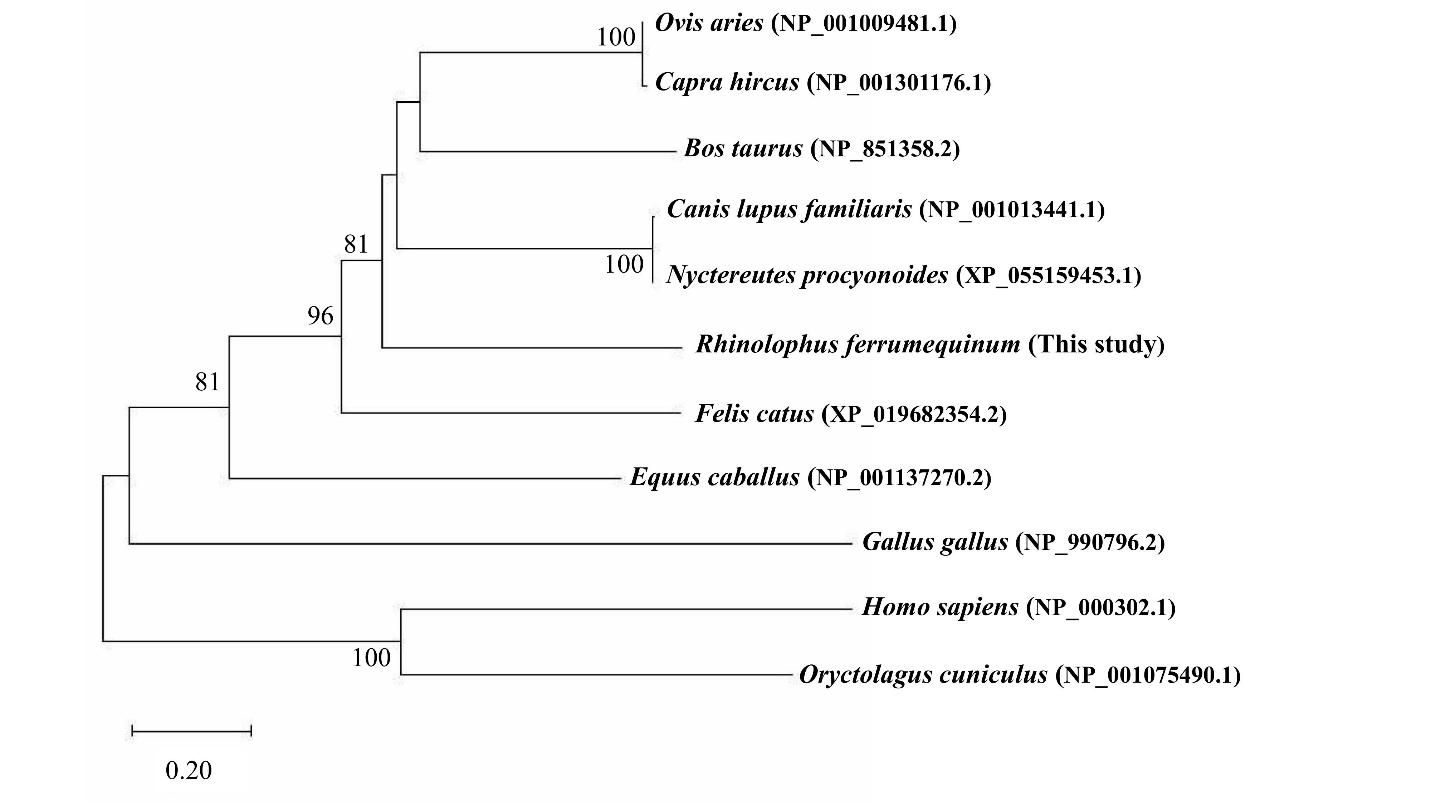
**

**Supplementary Figure S2.** The phylogenetic tree of *PRNP* amino acid sequences among bats and a diverse range of species. The tree was constructed using the Maximum Likelihood method with 100 bootstrap replicates, and bootstrap values (%) are indicated at the nodes.
